# *BORIS* (*CTCFL*) Is Not Expressed in Most Human Breast Cell Lines and High Grade Breast Carcinomas

**DOI:** 10.1371/journal.pone.0009738

**Published:** 2010-03-17

**Authors:** William C. Hines, Alexey V. Bazarov, Rituparna Mukhopadhyay, Paul Yaswen

**Affiliations:** 1 Life Sciences Division, Lawrence Berkeley National Laboratory, Berkeley, California, United States of America; 2 Department of Laboratory Medicine, University of California San Francisco, San Francisco, California, United States of America; National Institute on Aging, United States of America

## Abstract

BORIS (CTCFL) is the only known paralog of the versatile regulatory protein CTCF, a multifunctional DNA binding protein that mediates distinct gene regulatory functions involved in cell growth, differentiation, and apoptosis. Unlike CTCF, the expression of BORIS is normally restricted to specific cells in testes (the only cells where CTCF is not expressed), where it may play a role in reprogramming the methylation pattern of male germ line DNA. Frequent amplification of the 20q13.2 region, which contains the *BORIS* gene, and expression of *BORIS* transcripts in diverse human tumors and cell lines have led to the hypothesis that aberrant expression of BORIS may play a role in tumorigenesis by interfering with CTCF functions. However, recent studies using more quantitative methods indicate low frequency of *BORIS* expression in melanoma, ovarian, prostate, and bladder carcinomas. To investigate the relationship between chromosome 20q13 amplification and *BORIS* mRNA levels within breast cancer cell lines and tissues, we developed a quantitative RT-PCR assay to measure the levels of *BORIS* mRNA. Endpoint RT-PCR assays were also used to investigate the possible expression of alternatively spliced variants. Using multiple primer sets and controls, we found that neither mature *BORIS* transcripts nor spliced variants are commonly expressed at detectable levels in malignant breast cells or tissues, although endogenous *BORIS* transcripts can be induced in MCF-7 cells following 5-aza-2′-deoxycytidine treatment. In conclusion, in most breast cancer cells, endogenous BORIS is unlikely to be expressed at sufficient levels to interfere with CTCF functions. Thus it is improbable that aberrant BORIS expression plays a role in most human breast cancers.

## Introduction

BORIS, first described as “Brother Of the Regulator of Imprinted Sites,” or CTCF-like protein (CTCFL; NM_080618), is the sole known paralog of CTCF (CCCTC-binding factor; NM_006565) - a multifunctional DNA binding protein that uses different sets of zinc fingers to mediate distinct functions in regulation of gene expression. These functions include context-dependent promoter repression or activation, creation of modular hormone-responsive gene silencers, and formation of enhancer blocking elements (insulators) (reviewed in [1]–[3]). Recent evidence indicates that CTCF is involved in the global organization of chromatin, and “may be a heritable component of an epigenetic system regulating the interplay between DNA methylation, higher-order chromatin structure, and lineage-specific gene expression” [3]. Unlike CTCF, the expression of BORIS is normally restricted to specific cells in testes (the only cells where CTCF is not expressed), where it may play a role in reprogramming the methylation pattern of male germ line DNA [4].

The genomic organizations of the *BORIS* and *CTCF* genes, which are located on chromosomes 20q13.2 and 16q22.1 respectively, suggest that the two genes evolved from a gene duplication event during vertebrate evolution [5]. The amino acid sequences composing the two proteins' eleven zinc finger motifs are nearly identical, but the sequences at the amino- and carboxy- terminal ends diverge markedly. This likely provides the proteins with similar DNA binding specificities/affinities, yet distinct protein functions [4]. In fact, Sun and colleagues have recently demonstrated, using a DNA methylase-deficient cell model, that competition between BORIS and CTCF is a possibility when both proteins are present in equal amounts [6], a situation that may occur in certain cancer cells.

Aberrant expression of BORIS has been proposed to play a role in tumorigenesis [7]. The 20q13.2 region where the *BORIS* gene is located is commonly amplified in significant percentages of malignancies in a variety of organs, and may harbor one or more oncogenes [8]–[11]. Aberrantly expressed *BORIS* transcripts have also been reportedly detected in diverse human tumors and tumor-derived cell lines, including nearly all those derived from breast tissues [12]–[21]. Other reports indicate BORIS contributes to the promoter-specific demethylation and derepression of several cancer-testis (CT; a class of genes expressed normally in the testis, but activated in a wide range of tumor types) genes [15], [19], although BORIS expression by itself appears to be insufficient for the induction of CT gene expression [22].

Despite its relationship to CTCF and its location within a commonly amplified genomic region, recent findings in melanoma, ovarian, prostate, and bladder carcinomas [14], [20], [22] appear to controvert a broad tumorigenic role for BORIS. These studies found that *BORIS* transcript expression was not as frequent in primary melanomas (27%) [14], [20], [22] as originally estimated for melanoma cell lines (90%) [14], [20], [22], and when measured quantitatively, levels in tumors were not statistically different from those in normal prostate, bladder, and ovarian tissues [14], [20], [22]. While initiating a study to investigate the molecular mechanism(s) leading to the aberrant *BORIS* expression in breast cancers, we have obtained similar discordant results regarding the expression of *BORIS*. Using sensitive RT-PCR-based assays, employing multiple primer sets, we find that neither mature *BORIS* transcripts, nor spliced variants, are commonly expressed in malignant breast cells. Thus it is unlikely that aberrant BORIS expression plays a role in most human breast cancers.

## Results

### Nearly all breast cancer cell lines and tumors lack detectable levels of *BORIS* mRNA

**Figure 1:**
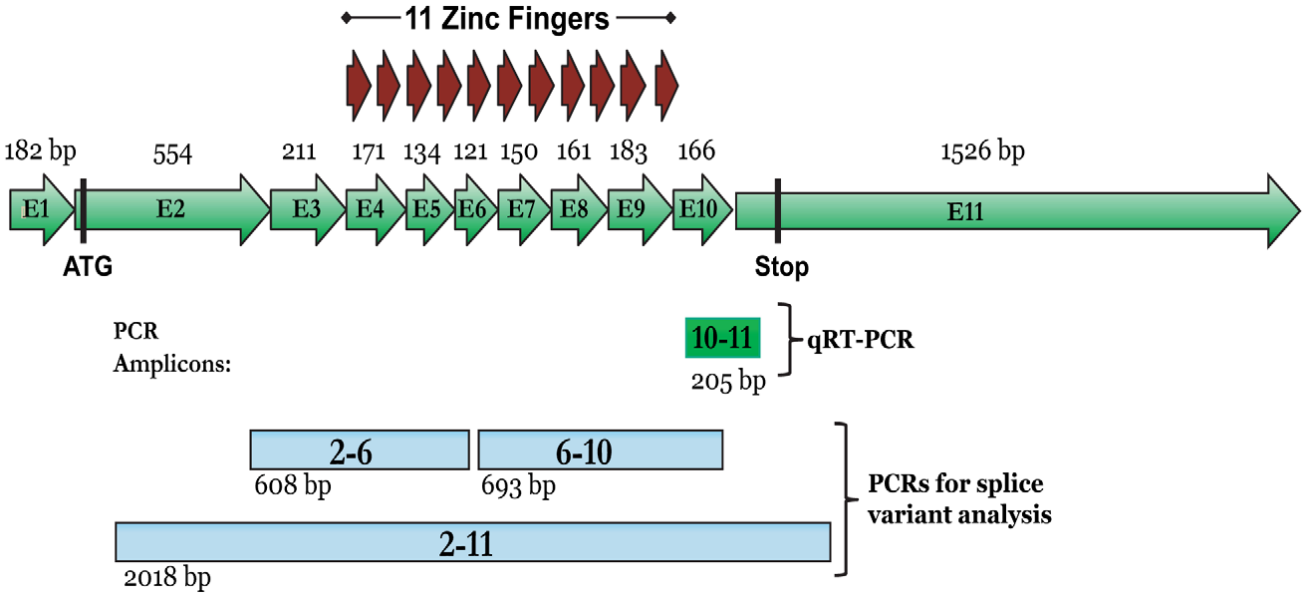
*BORIS* mRNA and PCR design. The eleven exons of *BORIS* mRNA (ENSEMBL# ENST00000243914, CTCFL), and their corresponding sizes (base pairs) are depicted as green arrows. The ATG start codon, area encoding the eleven zinc fingers (red arrows), and TGA stop codon are marked for reference. The areas and sizes of the real-time PCR amplicon (10-11; bright-green box), and end-point PCR amplicons (2–6, 6–10, 2–11; blue boxes) used to detect alternatively spliced variants, are indicated.

To investigate the relationship between chromosome 20q13 amplification and *BORIS* mRNA levels, we developed a quantitative RT-PCR assay (exons 10–11, Figure 1) to measure the levels of *BORIS* mRNA within breast cell lines having either normal or amplified 20q13 DNA regions [23]. Sensitivity and amplification efficiencies of the *BORIS, CTCF*, and *TBP* PCR reactions were determined by amplifying dilutions of plasmids containing the corresponding coding sequences (Table 1). The *BORIS* qRT-PCR assay remained linear down to 2.86 attograms (2.86^−18^ g) of plasmid DNA, equivalent to 24 copies of *BORIS*. Using the same assay, *BORIS* transcript levels were undetectable in control BJ fibroblasts; but in BJ fibroblasts transduced with CMV-HA-*BORIS* adenovirus, were over 32,000 times greater than those in testis (Table 1). Altogether, the results of these individual assays and controls established the specificity, sensitivity, and large dynamic range of the *BORIS* qRT-PCR assay.

**Table 1:** Detection of *BORIS* mRNA in breast cell lines, primary tumors, and controls.

Gene		BORIS	BORIS	BORIS	BORIS	TBP	
Exons		(2–11)	(2–6)	(6–10)	(10–11)		
Data Type		PCR band	PCR band	PCR band	Ct value	Ct value	BORIS (%TBP)
Category	Sample						
Cell Lines	MDA-MB-453	NP	**-**	**-**	ND	26.9	0
	MDA-MB-231	**-**	**-**	**-**	ND	25.72	0
	MCF-10A	NP	**-**	**-**	ND	26.52	0
	T47D	NP	**-**	**-**	ND	26.79	0
	MCF-12A	NP	**-**	**-**	ND	26.76	0
	MDA-MB-436	NP	**-**	**-**	37.71	26.17	0.033
	MCF-7*	-	**-**	**-**	ND	25.82	0
	SUM185PE*	NP	**-**	**-**	ND	26.91	0
	UACC812*	NP	**-**	**-**	ND	26.67	0
	AU565*	NP	**-**	**-**	ND	26.94	0
	BT474*	NP	**-**	**-**	ND	26.38	0
	SKBR3*	NP	**-**	**-**	ND	28.5	0
	MDA-MB-435†	NP	**+**	**+**	35.11	28.16	0.809
Breast							
Tumors	T686	NP	**-**	**-**	ND	28.59	0
	T688	NP	**-**	**-**	ND	31.39	0
	T689	NP	**-**	**-**	ND	28.99	0
	T691	NP	**-**	**-**	ND	29.9	0
	T693	NP	**-**	**-**	ND	28.1	0
	T694	NP	**-**	**-**	ND	27.85	0
	T695	NP	**-**	**-**	ND	29.78	0
	T-Amb	NP	**-**	**-**	ND	29.95	0
Normal Breast							
Tissues	N-Amb	NP	**-**	**-**	ND	29.9	0
	N697	NP	**-**	**-**	NP	#	0
Controls	Water	**-**	**-**	**-**	ND	ND	0
	minus RT	**-**	**-**	**-**	ND	ND	0
	genomic DNA	**-**	**-**	**-**	ND	ND	0
	Testes	**+**	**+**	**+**	27.35	24.03	9.151
	BORIS plasmids (dilution series)	**+**	**+**	**+**	18.47-35.34	NA	NA
	BJ Fibroblasts	NP	**-**	**-**	ND	24.07	0
	BJ + Adeno-BORIS	NP	**+**	**+**	13.09	24.62	2.96×10^5^

Three endpoint RT-PCR assays, spanning different intron splice junctions, were designed to detect mature transcripts and alternatively spliced variants of *BORIS* mRNA (2–11, 2–6, 6–10; Figure 1). These RT-PCR reactions were performed using RNA derived from cell lines, breast tumors, normal breast tissue, or several different positive and negative controls (described in text). Cell lines with evidence of 20q13.2 amplification [23] are indicated by an asterisk (*). The detection of *BORIS* mRNA using the different end-point PCR assays is indicated by a “+”, whereas the lack of a product is indicated by a “-”. NP  =  not performed. A quantitative real-time RT-PCR (qRT-PCR) assay, spanning intron 10 (10–11), was used to quantify *BORIS* mRNA levels. The median threshold cycle (Ct; the PCR cycle for which products are detected above baseline), of triplicate reactions is reported for both *BORIS* (10–11) and *TBP*. *BORIS* mRNA was not detected (ND) in most cell lines and all tumors. Quantitative results were normalized and are expressed as a percentage of *TBP* expression (% *TBP*). For the N697 sample, only *TBP* end-point PCR was performed and it was positive (#). MDA-MB-435 is considered to be melanoma-derived (†).

**Figure 2:**
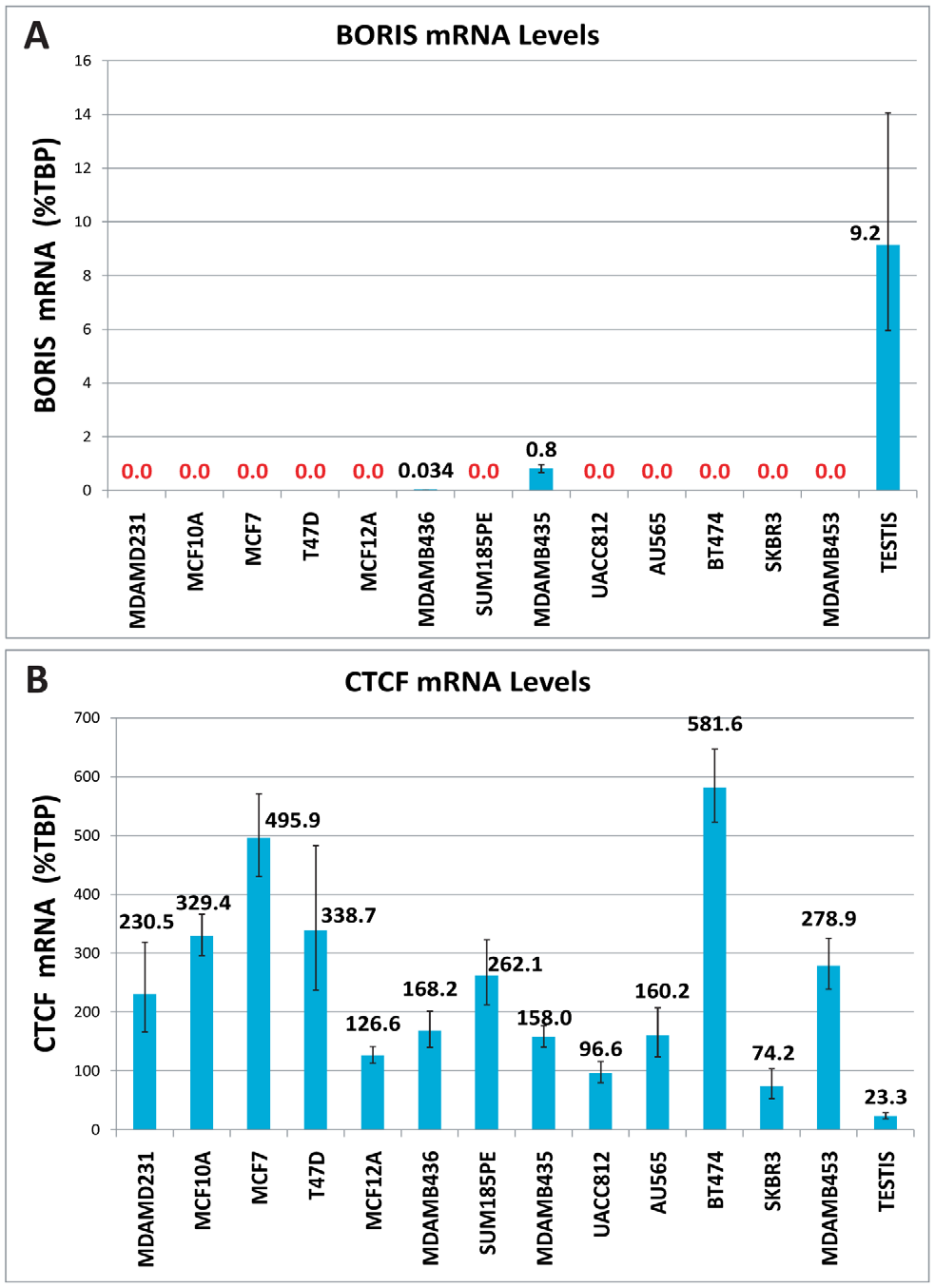
*BORIS* and *CTCF* mRNA levels in cell lines. Levels of (A) *BORIS* and (B) *CTCF* mRNA were measured by real time PCR in cell lines and testis (provided as a physiological control), and are reported relative to the levels of internal reference transcript *TBP*. *BORIS* mRNA was detectable in the breast cell line, MDAMB436, and in the melanoma-derived cell line, MDAMB435, but not in the remaining breast-derived cell lines. *CTCF* mRNA levels were near or above the levels of *TBP* mRNA in all cell lines. Testis expressed the lowest *CTCF* level, near the level of *BORIS* in this sample (note the differences in scale between the graphs).

Using this assay, we found evidence of *BORIS* expression in one breast cancer-derived cell line, MDA-MB-436, at a level (0.033% *TBP*) near the detection limit of 40 cycles (Figure 2A). A 24-fold higher level of *BORIS* (0.809% *TBP*) was detected, however, in the melanoma-derived MDA-MB-435 cell line. No evidence of *BORIS* expression was found in any of the remaining breast cell lines. Notably, neither MDA-MB-436 nor MDA-MB-435 exhibit 20q13 amplification (Table 1). Testis RNA was used as both a positive control and reference of physiological levels of *BORIS* in all experiments, and *BORIS* mRNA levels in testis were 272-fold higher than levels in the MDA-MB-436 cells. The successful amplification in all samples of *TBP* transcripts, used as internal controls, confirmed both the integrity and successful reverse transcription of the RNA (Table 1).

In addition to RT-PCR assays, we attempted to detect BORIS protein by immunoblot analyses; however, all of the commercially available antibodies that we tested were unable to detect BORIS protein in MCF-7, MDA-MB-231, or MDA-MB-435 cell lines infected with adenoviral *BORIS* expression vectors (data not shown). Virally mediated *BORIS* mRNA expression was confirmed in all three infected cell lines (7.1, 5.1, and 1.1% of the level of *TBP* transcripts, respectively).

To investigate the prevalence and levels of *BORIS* expression within breast tumors, samples of eight unrelated tissue specimens were analyzed by qRT-PCR. All tumors were high grade (grade III), invasive ductal carcinomas. *BORIS* transcripts were not detected in any breast tumor samples (Table 1). In addition, two non-malignant breast samples were also analyzed, and did not express detectable levels of *BORIS* transcripts (Table 1).

### An alternatively spliced variant of *BORIS*, missing exon 7, is present in testis, but is absent in breast cancer cell lines and tumors

**Figure 3:**
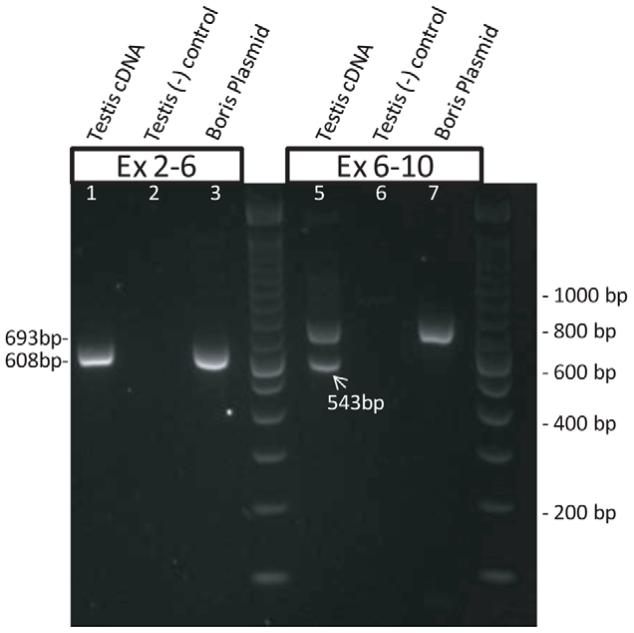
Expression of an alternatively spliced variant of *BORIS* in testis. BORIS specific primers, designed to amplify the region of *BORIS* between exons 2–6 (lanes 1–3) and exons 6–10 (lanes 5–7), were used to screen for possible alternatively spliced variants of *BORIS*. The expected sizes of these two products, based on the published sequence of mature *BORIS* mRNA, are 608 bp and 693 bp, respectively. These full-length products were present when testis cDNA (lane 1 & 5) and a plasmid containing a full length *BORIS* cDNA (lane 3 & 7) were used as PCR templates. In testis, an additional band (543 bp) was also amplified by the exon 6–10 PCR primers (lane 5). PCR products were not present in the testis (-RT) negative control reactions (lanes 2 & 6).

To investigate the possibility that alternatively spliced forms of *BORIS*, undetectable by the above qRT-PCR assay, were being expressed in breast cell lines and tissues, we developed three endpoint RT-PCR assays (Figure 1). The primers were designed to detect all published *BORIS* transcripts, including those generated through the use of alternative promoters [24]. Using these assays and the above-described controls, we found, in agreement with the qRT-PCR data, that *BORIS* transcripts were detectable in MDA-MB-435, testis, and *BORIS*-transduced fibroblasts. *BORIS* expression was not detectable in any of the remaining breast cell lines or tissues (Table 1). A splice variant was present, however, in testis, as indicated by two bands in both the Exon 2/11 (data not shown) and Exon 6/10 PCR reactions (Figure 3); these two variants were roughly equal in abundance. Variants were not detected using the Exon 2/6 primers (Figure 3). The Exon 6/10 products were isolated from agarose, cloned into the pGEM® T-Easy T/A vector (Promega, Madison, WI), and analyzed by DNA sequencing. The sequence of the larger product corresponded to the expected full-length *BORIS* sequence located between the assay's PCR primers. The smaller product was identical, except that it lacked 150 bases found in the larger product, in accordance with its estimated size determined from the agarose gel. The missing sequence was identified to be the entire seventh exon of *BORIS*.

### *BORIS* expression is detected in MCF-7 cells following 5-aza-2′-deoxycytidine treatment

**Figure 4:**
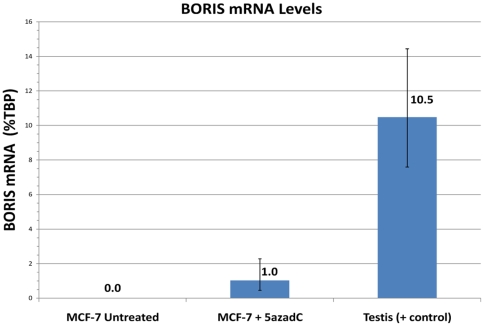
Endogenous *BORIS* mRNA expression is induced by 5-aza-2′-deoxycytidine. *BORIS* mRNA levels in MCF-7 cells were determined by qRT-PCR (as in Figure 2). Endogenous *BORIS* mRNA was detectable (1.02% *TBP*) only after treatment with the demethylating agent, 5-aza-2′-deoxycytidine (5-aza-dC) and at a level approximately one tenth of that in testis (1.02% vs. 10.47% *TBP*).

Treatment of cells with the demethylating agent, 5-aza-2′-deoxycytidine (5-aza-dC), has been previously demonstrated to result in transcriptional activation and elevated levels of *BORIS* mRNA [14], [15], [19], [20]. To determine if similar effects occur in breast cells (as well as to test our assay's ability to detect endogenous *BORIS* transcripts), we evaluated *BORIS* mRNA levels by qRT-PCR in MCF-7 cells following exposure to 10 µM 5-aza-dC (72 hr exposure, followed by 24 hrs in growth medium without 5-aza-dC). This treatment of MCF-7 cells resulted in an increase in *BORIS* transcripts from undetectable levels to levels roughly equivalent to those in MDA-MB-435 melanoma cells (1.02 vs. 0.81%TBP), about 10% of the levels present in testis (Figure 4).

### *CTCF* mRNA is abundant in breast cancer cell lines

Aberrant BORIS expression is currently hypothesized to contribute to tumorigenesis by competing with CTCF for DNA binding sites. To evaluate the feasibility of this hypothesis, we measured *CTCF* mRNA levels in the breast tumor-derived cell lines (Figure 2B). *CTCF* transcripts were detected within all cell lines (and the testis control). Levels of *CTCF* mRNA ranged from 75–581% of *TBP* levels in the cell lines. Notably, testis exhibited the lowest level of *CTCF* transcripts, nearly equivalent to the level of *BORIS* mRNA in this sample (23.3 vs. 9.2% of *TBP*).

## Discussion

In the course of investigating the relationship between chromosome 20q13 amplification and its effects on *BORIS* expression, we have found only a single cell line among 12 breast cancer cell lines and 8 tumors to express detectable levels of *BORIS* mRNA. We therefore conclude that the presence of 20q13 amplification is insufficient to derepress *BORIS* transcription, and that aberrant *BORIS* expression in breast cells is not a prevalent factor facilitating or maintaining their malignant character.

These results were unexpected, given claims of *BORIS* expression in a large number of tumors and its absence in associated somatic tissues [7], [19], [21]. To investigate this discordance, we re-evaluated both the specificity and sensitivity of our qRT-PCR assay using several controls. Specificity has been demonstrated by cloning and sequencing PCR products, by detecting *BORIS* transcripts in cells transduced with a *BORIS*-encoding adenovirus, and by the lack of PCR products in all negative controls (-RT, water, genomic DNA). Sensitivity of the assay has been demonstrated by amplifying log dilutions of a *BORIS*-containing plasmid. In addition, we were able to successfully detect endogenous *BORIS* mRNA in testis, two (breast- and melanoma-derived) cell lines, as well as in MCF-7 cells treated with 5-aza-dC.

We also performed PCR assays designed to detect possible splice variants that, if expressed in place of full-length *BORIS*, would lead to false-negative results. No such variants were determined to be present in any of the breast tissues or cell lines. However, a splice variant was detected in the testis, and it was expressed at approximately the same level as the full-length *BORIS* mRNA. This variant, which lacks exon 7 (encoding part of the zinc finger region), may have an important function in the testis, and its presence warrants further investigation.

Our results are inconsistent with those of Vatolin and colleagues, who reportedly detected expression of *BORIS* by endpoint RT-PCR in 7/8 breast cell lines and 11/12 breast tumors using PCR primers that span exons 3–10 [19]. Five of these eight cell lines are common to this study: T47D, MDA-MB-231, MDA-MB-453, MCF-7, and MDA-MB-435. Notably, the region amplified in our exon 6–10 PCR reactions was flanked by the same reverse primer sequence used by Vatolin *et al*. [19]. Therefore, the results of these two assays should have corresponded. Furthermore, we did not detect *BORIS* transcripts in any of the eight primary breast tumors we investigated, all of which were grade III tumors. Histopathological information was not provided in the original publication by Vatolin *et.al*., precluding a comparison of the tumor stage/grade in the two studies. In a more recent publication, this group reported that both *BORIS* mRNA and protein were detectable in multiple invasive breast carcinomas, although again stage and grade were not specified [7], [19], [21].

Overall, we did not detect *BORIS* mRNA in 6 of the 7 breast cell lines determined to be positive by Vatolin *et.al.* [19]. However, in accordance with their results, we did demonstrate *BORIS* mRNA expression in MDA-MB-435 cells and lack of expression in MCF-7 cells. The later report from this group [21] that MCF-7 cells express BORIS protein is in apparent conflict with this result. Our finding that 5-aza-dC treatment resulted in the activation of *BORIS* expression in MCF-7 cells is also consistent with previous findings. Of the two cell lines that we did find to be *BORIS*-positive, MDA-MB-435 cells expressed the highest levels of *BORIS* transcripts. However, although previously believed to be a breast cell line, MDA-MB-435 is currently regarded as a derivative of the malignant melanoma cell line M14 [25], [26]. This is notable due to the frequent expression of CT genes in melanomas; e.g., MAGEA1 (reviewed in [27]). Thus, the melanoma origin of MDA-MB-435 may explain why *BORIS* expression was found in this cell line.

We have determined and described both the specificity and sensitivity of our assays, and cannot account for these discrepant results other than to suggest that the levels described by Vatolin *et.al*. may have been lower than our threshold of detection. It is possible that the high number of PCR cycles used by Vatolin *et al*. (40–45 cycles) led to the detection of faint *BORIS* expression, possibly from subpopulations of cells. Based on their endpoint RT-PCR data, however, *BORIS* and *CTCF* transcripts appeared to be expressed at roughly equivalent levels in the breast cell lines. This contrasts with our qRT-PCR data in which we saw robust *CTCF* expression but undetectable *BORIS* levels in most cell lines and tissues. Although one other study has reported that BORIS protein is expressed in MDA-MB-231 cells [7], [19], [21], we were unable to detect *BORIS* mRNA in this cell line. In addition, using the same commercial antibody, we were unable to confirm the expression of BORIS protein in MDA-MB-231 cells. We cannot account for these discrepant findings, other than to suggest that variations in the cell line might be responsible. Nevertheless, our general conclusion is supported by other studies that have used serial analysis of gene expression (SAGE) to investigate aberrant gene expression in breast tumors and cell lines [28], [29]. These SAGE studies did not report the expression of *BORIS* mRNA in the samples analyzed. Notably, the study by Yao et al. [28] was specifically designed to identify differentially expressed genes encoded within commonly amplified chromosomal regions, including the 20q13 region where the *BORIS* gene is located.

Aberrant BORIS expression has been proposed to facilitate cell transformation by competing for CTCF binding sites, leading to disruption of insulator boundaries and abnormal gene expression [7]. For a competition model to be feasible in breast cancer, levels of *BORIS* and *CTCF* mRNA and/or protein would have to be comparable. We found however, that while *CTCF* mRNA was quite abundant in breast cells, *BORIS* mRNA was generally below our detection limit. Even in the MDA-MB-468 breast cell line, in which *BORIS* transcripts were detectable, levels of *CTCF* mRNA were, on average, at least 9000-fold higher than those of *BORIS* mRNA. At these disparate levels, it is unlikely that differences in protein stability would make sufficient BORIS protein available to compete with CTCF at DNA binding sites. Thus, aberrant BORIS expression is unlikely to compete with CTCF, even in the rare breast cancer cell line in which it is expressed.

## Materials and Methods

### Tissue samples

Frozen breast tissue specimens (seven malignant, and one reduction mammoplasty sample) were provided by the Cooperative Human Tissue Network. The tumor specimens were grade III (Scarf-Bloom-Richardson) invasive ductal carcinomas. Individual samples of RNA isolated from non-malignant and malignant breast, as well as testis, were purchased from Ambion (Austin, TX).

### Cell culture

Normal BJ fibroblasts and the breast cancer-derived cell lines: MDA-MB-453; MDA-MB-231; T47D; MDA-MB-436; MCF-7; UACC-812; AU-565; BT474; and SK-BR-3; the non-malignant derived cell lines: MCF-10A and MCF-12A; as well as the MDA-MB-435 cell line, which is currently regarded to be a derivative of the M14 malignant melanoma cell line (and will be referred to as such herein) [25], [26], were obtained from the American Type Culture Collection. Another breast cancer cell line, SUM185PE, was provided by Dr. Joe Gray (LBNL). Cells were propagated and subcultured using conditions indicated by the supplier.

### *BORIS* adenovirus

To create the recombinant adenovirus, a 3483 bp NheI/NotI DNA fragment, containing the *BORIS* coding region, was subcloned from pBIG2i-HA *BORIS* (generously provided by V. Lobanenkov, NIH) [15]. This fragment was ligated into an NheI/Not I linearized pShuttle vector (Adeno-X™ Expression System, Clontech). Generation of recombinant adenovirus and virus particles were completed using the manufacturer's protocol. This adenovirus contains the coding sequence of HA-tagged *BORIS*, verified by DNA sequencing, under control of the CMV promoter. Adenovirus titers were determined by immunocytochemical detection of the adenovirus hexon protein in infected HEK293 cells (Adeno-X™ Rapid Titer, Clontech). This virus was used to infect BJ fibroblasts using 10 infection-forming units per cell. Functional activity of the *BORIS* adenovirus was confirmed by qRT-PCR using RNA isolated from transduced cells.

### RNA isolation and reverse transcription

Total RNA was isolated using silica-based spin-column extraction kits (RNeasy mini kit, Qiagen) using the manufacturer's protocol. Total RNA was treated with DNA-free DNAse I (Ambion) to reduce contaminating DNA. RNA integrity was evaluated by agarose gel electrophoresis using Gel Star (Cambrex) nucleic acid gel stain. Complimentary DNA (cDNA) was synthesized from 2 µg of total RNA by random-decamer primed reverse transcription using the Retroscript reverse transcription kit (Ambion) and the manufacturer's standard protocol. Negative (-RT) controls contained RNase-free water substituted for reverse transcriptase.

### Quantitative real-time PCR

*BORIS* and *CTCF* mRNA levels were measured in breast tissue specimens and cell lines using the Platinum® Quantitative PCR Supermix (Invitrogen). The sequences of forward primers, reverse primers, and Taqman probes for the systems are described in Table 2. Both transcripts were amplified in parallel, along with a stably expressed reference gene, *TBP* (TATA box binding protein; NM_003194), in triplicate reactions, from equal amounts of cDNA (1 µl of the reverse transcription reaction). The qRT-PCR *BORIS* primers amplify a 205 base pair (bp) sequence spanning the splice junction between exons 10 and 11, flanking a 4734 bp intron. *CTCF* mRNA levels were measured by SYBR Green qRT-PCR (SYBR Greener™, Invitrogen), using the same conditions and controls described for the *BORIS* qRT-PCR assay. The *CTCF* primers amplify a 135 bp sequence element and flank a 3818 bp intron. The final concentrations were 300 nM of each forward and reverse primer, and 100 nM of probe, when used. PCR amplification was performed using the Biorad MyiQ Single-Color Real-Time PCR Detection System using the following standard amplification protocol: 50°C×2 min, 95°C×2 min, 40 cycles: 95°C×15 sec, 60°C×60 sec. Testis cDNA and plasmid DNA containing the *BORIS* coding sequence were used as positive controls, as was cDNA from fibroblasts transduced with the *BORIS*-containing adenovirus. Water and –RT reactions were both used as negative controls to detect possible amplification from contaminating DNA. The identities of the PCR products were confirmed by gel analyses and DNA sequencing. Amplification efficiencies, determined by amplifying log dilutions of plasmids containing the corresponding coding sequences, were determined to be near 100%. Relative levels of *BORIS* and *CTCF* transcripts were calculated using the delta Ct method and normalized to those of the *TBP* reference transcript using the formula: %TBP  = 2^−(Ct (BORIS)−Ct (TBP))^×100%. Standard deviations of triplicate reactions were used to propagate error using the square root of the sum of squares method.

**Table 2:** Quantitative RT-PCR primer and probe sequences.

BORIS, Exon 10/11 (qPCR)	For	5′- ACCTGCACAGACATTCGGAGAAGT
	Rev	5′- AACTGTTCTCCCTTCGTGGTGGAA
	Probe	5′- 6FAM-TTCCCTTTCCTGAAGCAGCCGACTTTGC-BHQ
BORIS, Exon 2/11	For	5′- TGTGCAGAGAGAAAGACCATCGGA
	Rev	5′- GCAGTGAACATGCAACCTGACTCT
BORIS, Exon 2/6	For	5′- TGGTGGCCAGTGAAGACAGTAAGT
	Rev	5′- GGATCGGACATGGCGCTTCAATTT
BORIS, Exon 6/10	For	5′- CTTTCAGTGTTGCCAGTGCAGCTA
	Rev	5′- TTCTGACCCTTTGTGGCTTCCTTC
CTCF	For	5′- TCGTCGTTACAAACACACCCACGA
	Rev	5′- CTGCACAAACTGCACTGAAACGGA
TBP	For	5′- CACGAACCACGGCACTGATT
	Rev	5′- TTTTCTTGCTGCCAGTCTGGAC
	Probe	5′- 6FAM-TGTGCACAGGAGCCAAGAGTGAAGA-BHQ

### Splice variant screening by conventional PCR

To detect the possible presence of alternatively spliced *BORIS* transcripts, three end-point PCR reactions were developed (Figure 1). Primer pairs were designed to amplify regions of *BORIS* mRNA between: 1) exons 2 and 11, 2) exons 2–6, and 3) exons 6–10. Reactions were performed using SYBR® GreenER PCR reagent (Invitrogen) and the Biorad PCR Detection System. The amplification parameters for these PCR assays were: 1) 50°C×2 min, 95°C×8.5 min, followed by 45 cycles 95°C×30 sec, 58°C×30 sec, 72°×2.5 min; 2) and 3) 50°C×2 min, 95°C×8.5 min, followed by 45 cycles 95°C×15 sec, 60°C×30 sec, 72°×1.5 min. Reaction products were analyzed as described above.

### Immunoblot analysis

BORIS protein levels were analyzed in MCF-7, MDA-MB-231, and MDA-MB-435 cell lines. To serve as positive controls, each of these cell lines was infected with the *BORIS* adenovirus, and the resulting expression of *BORIS* mRNA was confirmed by qRT-PCR. Parallel cell cultures were lysed in RIPA buffer (50 mM Tris•HCl pH 8.0, 150 mM NaCl, 1% NP-40, 0.5% Na Deoxycholate, 0.1% SDS) containing protease (Protease Inhibitor Cocktail Set III EDTA-free, Calbiochem) and phosphatase (Phosphatase Inhibitor Cocktail Set II, Calbiochem) inhibitors, both diluted 1∶100. The lysates were incubated on ice for 15 minutes, then centrifuged 24,000×g for 30 min at 4°C. Twenty-five micrograms of each lysate were loaded per well onto 4–12% gradient polyacrylamide gels (NuSep). After electrophoresis, the proteins were transferred to nitrocellulose, and probed with antibodies using standard conditions. Three anti-Boris antibodies were tested: Abcam (Ab18337, lots 123956 and 469726, 1∶2500 dilution), Rockland (600-401-907, lot 21606, 1∶1000 dilution), and Sigma (HPA001472, lot A08951, 4 ug/ml final concentration). The blots were probed with anti-β-actin antibodies (Sigma, A1978, lot 118K4827) to control for loading differences. Signals were imaged using an Odyssey infrared imaging system (Licor Biosciences).
